# Illustrating a Species Sensitivity Distribution for Nano‐ and Microplastic Particles Using Bayesian Hierarchical Modeling

**DOI:** 10.1002/etc.5295

**Published:** 2022-02-28

**Authors:** Kazutaka M. Takeshita, Yuichi Iwasaki, Thomas M. Sinclair, Takehiko I. Hayashi, Wataru Naito

**Affiliations:** ^1^ Health and Environmental Risk Division National Institute for Environmental Studies, Tsukuba Ibaraki Japan; ^2^ Research Institute of Science for Safety and Sustainability National Institute of Advanced Industrial Science and Technology, Tsukuba Ibaraki Japan; ^3^ Department of Animal and Plant Sciences University of Sheffield Sheffield United Kingdom; ^4^ Social Systems Division National Institute for Environmental Studies, Tsukuba Ibaraki Japan

**Keywords:** Aquatic organisms, Bayesian estimation, Ecotoxicity, Particle size, Polymer type, Test type of medium

## Abstract

Environmental contamination with nano‐ and microplastic (NMP) particles is an emerging global concern. The derivation of species sensitivity distributions (SSDs) is an essential step in estimating a hazardous concentration for 5% of the species (HC5), and this HC5 value is often used as a “safe” concentration in ecological risk assessment, that is, predicted‐no‐effect concentration. Although properties of plastics such as particle size can affect toxic effect concentrations, such influences have not yet been quantitatively considered in estimating SSDs for NMP particles. We illustrate a log‐normal SSD using chronic lowest‐observed‐effect concentrations (LOECs) of NMP particles from readily available toxicity data sets, considering the influence of particle size, polymer type, and freshwater or marine test media by adopting Bayesian hierarchical modeling techniques. Results of the hierarchical SSD modeling suggest that the SSD mean was negatively associated with particle size and was lower in marine media than in freshwater media. The posterior medians of the HC5 estimated from the LOEC‐based SSD varied by a factor of 10 depending on these factors (e.g., 1.8–20 μg/L for the particle size range of 0.1–5000 μm in the marine environment). Hierarchical SSD modeling allows us to clarify the influences of important factors such as NMP properties on effect concentrations, thereby helping to guide more relevant ecological risk assessments for NMP. *Environ Toxicol Chem* 2022;41:954–960. © 2022 The Authors. *Environmental Toxicology and Chemistry* published by Wiley Periodicals LLC on behalf of SETAC.

## Graphical abstract

Deriving species sensitivity distributions for nano‐ and microplastic particles that quantitatively incorporate the influence of particle size, polymer type, and freshwater or marine test media on toxic effect concentrations.

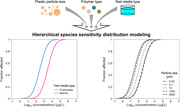

## INTRODUCTION

Marine and freshwater contamination with small plastic particles (nano‐ and microplastics; NMPs) is an emerging concern worldwide (Everaert et al., 2020; Koelmans et al., 2017). Massive research efforts are underway to understand and quantify the environmental fate, exposure, and risks and impacts of NMP particles (Adam et al., 2019; Besseling et al., 2019; de Ruijter et al., 2020; Everaert et al., 2020; Gouin et al., 2019; Koelmans et al., 2020). Despite several risk assessments having concluded that the ecological risks of microplastics are currently not of global concern except at a few hotspots (Adam et al., 2021; Everaert et al., 2020; Koelmans et al., 2020), important issues such as consideration of relevant effect mechanisms and the influences of the NMP properties (e.g., particle size and shape and polymer type) still need to be resolved to perform more defensible ecological risk assessments.

The application of a species sensitivity distribution (SSD) to toxic effect concentrations obtained from laboratory toxicity tests is a crucial step in deriving predicted‐no‐effect concentrations (PNECs) in ecological effect assessments of chemicals (Posthuma et al., 2002, 2019). Although several SSDs have been derived for NMPs (Adam et al., 2019; Besseling et al., 2019; Everaert et al., 2020; Koelmans et al., 2020), they were largely based on available toxicity data without quantitatively considering the influences of NMP properties or by assuming a relevant mode of action (“food dilution”); the latter recently proposed approach is a promising way to align exposure and effect assessments (Koelmans et al., 2020). In addition, previous relevant studies examined only the influences of NMP properties for single species (see Adam et al., 2021; Yang & Nowack, 2020) or discussed them qualitatively (Besseling et al., 2019). Although no attempt has been made so far, SSDs that quantitatively and simultaneously consider the influences of NMP properties can be derived by employing an approach of hierarchical modeling with Bayesian parameter estimation techniques (Hayashi & Kashiwagi, 2010; Kon Kam King et al., 2015). Such a statistical modeling approach would be valuable for examining how effect concentrations for NMPs, which are expressed by, for instance, the mean and standard deviation of a log‐normal SSD, are affected by their properties.

In the present study, we aimed to derive a log‐normal SSD for NMPs by applying hierarchical modeling techniques (hierarchical SSD [HSSD]) to available effect data acquired directly from Besseling et al. (2019). By doing so, we also examined the influence of two properties of NMPs (particle size and polymer type) and test type of medium (i.e., marine or freshwater) on effect concentrations of NMPs. Finally, using the derived SSD for NMPs, we derived the hazardous concentrations for 5% of species (HC5) considering the influences of these factors quantitatively. Given that the number of studies reporting effects of NMPs has been rapidly growing and the quality criteria for microplastic effect studies have been recently published (de Ruijter et al., 2020), use of updated effect data sets is ideal to derive a more scientifically robust SSD and rigorously examine the influences of those factors, which is not an easy task. However, we believe that, as an example, illustrating the HSSD approach based on the readily available effect data sets in the present study is valuable as a timely support of its application to ecological risk assessments for NMPs.

## MATERIALS AND METHODS

### Data

To derive HSSDs considering the properties of NMP particles, we collected chronic lowest‐observed‐effect concentrations (LOECs) of NMP particles for aquatic organisms directly from Besseling et al. (2019). Those authors compiled the effect concentrations of NMP particles in freshwater and marine media and converted the reported effect concentrations (e.g., median lethal concentration, median effect concentration) that were based on the endpoints of survival, reproduction, and growth to chronic LOEC values using the extrapolation factors proposed by Diepens et al. (2016). The extrapolation factors for deriving chronic LOECs ranged from 1 to 30 and were determined based on the type of effect concentration and the exposure duration for each effect data (see Besseling et al. [2019] and Diepens et al. [2016] for more details). In addition, information on the conditions of individual toxicity tests (e.g., type of medium, polymer type, particle size, and particle shape) is summarized in the data sets available in Besseling et al. (2019). It should be emphasized that we did not perform any further quality evaluation given that the primary aim of the present study is to illustrate the HSSD modeling for NMP particles. When two or more effect concentrations were available under the same test conditions (i.e., particle size, polymer type, and type of medium) for one species, we used their geometric mean values for the HSSD modeling (see the section *Implications for future model development and application* for more details about considering the intraspecies variations in effect concentrations). In the present study, effect concentrations obtained from tests with spherical NMP particles were used for the HSSD derivation to minimize the influence of particle shape and because the effect data for the other shapes (fiber and irregular) were limited (11% of all available data in Besseling et al. [2019]). Also, although six LOECs were categorized as brackish in Besseling et al. (2019), we categorized five of them as tested in marine media and the LOEC for *Hyalella azteca* as tested in a freshwater medium based on the original articles. A total of 26 LOECs that included 16 species across eight phyla (Chordata, Arthropoda, Mollusca, Rotifera, Echinodermata, Magnoliophyta, Chlorophyta, and Ochrophyta) were used for the HSSD modeling. Of the 26 LOECs, 10 and 16 were tested in freshwater and marine media (salinity of the seawater used [mostly] ~30‰), respectively; and a large portion (~90%) of them were based on growth and/or survival endpoints.

### HSSD model

In the present study, hierarchical modeling techniques were used to derive SSDs that accounted for the influences of particle size, polymer type, and/or type of medium on the chronic LOEC values of NMP particles. The HSSD models were expressed by the following equations:

(1) $$\log_{10}\text{LOEC}\sim \text{Normal}\left(\mu ,\sigma \right)$$

(2) $$\mu =\alpha +\sum \beta_{i}\left(X_{i}\right)+r_{j}$$

(3) $$r_{j}\sim \text{Normal}(0,\sigma_{\text{Ref}})$$

In these equations, LOEC is the chronic LOEC; *μ* and *σ* are the mean and standard deviation of the normal SSD, respectively; α is the intercept; *β*_*i*_ is the coefficient for the *i*th predictor variable *X*_*i*_, possibly associated with the mean value (i.e., *μ*) of the chronic LOECs (*β*_size,_ *β*_media,_ and *β*_polymer_); and *r*_*j*_ is the reference‐level random effects (reference *j*). Details about the predictor variables and the reference‐level random effects are described next. We assumed that *r*_*j*_ followed a Gaussian distribution with a mean of 0 and standard deviation of *σ*_Ref_.

As a predictor variable, the log_10_‐transformed plastic particle diameter (micrometers) was used to consider the influence of particle size on the mean of the SSD (i.e., *μ*). If a range of particle sizes was only available for a single effect concentration, we calculated the arithmetic mean value from the maximum and minimum particle sizes. The resulting range of particle sizes was 0.04–315.0 μm. In addition, a binary dummy variable corresponding to type of medium (freshwater, 0; marine, 1) was used as a predictor variable to consider the influence of type of medium (Wheeler et al., 2002). Furthermore, to consider the influence of polymer type of NMP particles, we categorized them as (1) polystyrene and mixtures of polystyrene and polyethyleneimine (*n* = 18 and 4, respectively; hereafter, PS) or (2) other polymer types (polyethylene, *n* = 3; polyvinyl chloride, *n* = 1). This is because the numbers of individual polymer types, excluding polystyrene, were very limited and because the difference in effect concentrations between polystyrene and non‐polystyrene was detected (Yang & Nowack, 2020). We used a binary dummy variable corresponding to the two polymer types (polymer types other than PS, 0; PS, 1) as a predictor variable. Because of the limited data availability, we assumed that the standard deviation of SSD was not affected by these properties in our HSSD modeling, but further study is required to test this assumption. We did not explicitly model the influences of the modes of action of NMP particles in the HSSD models; however, it can be interpreted that such influences were indirectly modeled by considering the influences of the NMP particle properties on effect concentrations.

Some reference (or test)–specific unmodeled factors may also influence the effect concentrations. For example, previous studies noted the variations in effect concentrations due to other properties of NMP particles, including origin (pristine particles or particles collected in the field) and removal of sodium azide (NaN_3_) stabilizer for nanoplastic particles (Besseling et al., 2019; Yang & Nowack, 2020). Thus, we included the reference‐level random effects (*r*_*j*_) in the HSSD models to consider such influences that could not be captured by the three predictor variables of particle size, polymer type, and type of medium.

### Parameter estimation, model selection, and estimation of HC5 values

Parameter estimation of the hierarchical models was conducted using a Bayesian framework. Posterior samples of the parameters were obtained using Hamiltonian Monte Carlo (HMC) sampling. Stan (Carpenter et al., 2017) with R 3.4.4 (R Core Team, 2018) and the package “rstan,” Ver. 2.18.2 (Stan Development Team, 2018) were used to conduct the HMC sampling. The two parameters for standard deviation (i.e., *σ* and *σ*_Ref_) were lower‐censored at zero. Two types of uniform distributions, the default settings of Stan, were set as the noninformative prior distributions of individual parameters: uniform distributions ranging from −∞ to ∞ for *α* and *β* and uniform distributions ranging from 0 to ∞ for *σ* and *σ*_Ref_. The R and Stan code for the HSSD model with all the predictor variables is available in the Supporting Information. We ran three chains in parallel with a burn‐in of 20,000 samples, which were discarded, followed by 10,000 samples that were thinned to retain every 10th sample, resulting in a total of 3000 samples as the posterior distributions of individual parameters. Convergence of sampling was assured with the criterion that the Gelman‐Rubin statistic $\hat{R}$ was <1.1 (Gelman et al., 2014).

Based on all possible combinations of the three predictor variables, we developed a total of eight candidate HSSD models. To rank the candidate models, we calculated the widely applicable information criterion (WAIC; Watanabe, 2010) values using the R package “loo,” Ver. 2.3.1 (Vehtari et al., 2018). The model with a smaller WAIC value has the higher predictive power for the dependent variable value (i.e., the log_10_‐transformed chronic LOECs) among the models evaluated, but a small difference in WAIC values indicates similar predictive powers between models. However, there is no theoretical criterion for the cutoff value for the difference in WAIC values, and cutoff values differ greatly among studies. Thus, in the present study, we selected the model with the minimum WAIC value as the best model and discuss results of the model selection considering other competitive models. Note that WAIC is not a metric reflecting the goodness of fit for the data analyzed.

We then obtained the SSD curves using the HMC samples of parameters of the best model and derived the posterior distribution of HC5. In the estimation of HC5, we did not incorporate the reference‐level random effects (*r*_*j*_) in the derivation of *μ* because our primary interest was to estimate the HC5 value unaffected by reference‐specific unmodeled factors. However, we also examined the influence of the random effects on the model predictions. Note that because the HSSD was estimated based on LOECs in the present study, the resulting HC5 values should be less conservative that those based on no‐observed‐effect concentrations but valuable to assess the likely ranges of “safe” concentrations.

## RESULTS AND DISCUSSION

### Overview of HSSD model selection

Among the eight candidate models, the HSSD model with two predictors of particle size and type of medium had the minimum WAIC value (Table 1; see also Supporting Information, Figure S1, for the relationship between observed and predicted LOECs). The plastic particle size was negatively associated with *μ* (i.e., the posterior median of the parameter *β*_size_; Table 2) in the best model, indicating that chronic LOEC values decreased with increasing particle size in this model. Similarly, the fact that the posterior median of the coefficient for type of medium (*β*_media_ in Table 2) was negative in the minimum WAIC HSSD model indicates that the chronic LOEC values in marine environments were lower than those in freshwater environments in this HSSD model. It should be noted that the 95% Bayesian credible intervals of the posterior distributions for particle size and type of medium included zero, such that further examination is required to reach more informative conclusions. The best model did not include polymer type, although the median coefficient (and 95% Bayesian credible interval) for polymer type in the second ranked model was –1.09 (–2.82 to 0.64; see Supporting Table S1). It is impossible to conclude which HSSD models are unsuitable for hazard assessment of NMPs based on WAIC values, but model selection results showed that the null model (i.e., with no predictor variables) was ranked fifth. This at least implies that the null SSD model is not the first choice to estimate an HC5 in terms of the predictive power, indicating the importance of including the properties of NMP particles, such as particle size, and type of medium as predictor variables.

**Table 1:** Results of model selection for the eight hierarchical species sensitivity distribution models with different combinations of the three predictor variables (particle size, type of medium, and polymer type)

Model rank	Particle size	Medium type	Polymer type	WAIC	ΔWAIC
1	+	+		68.77	0.00
2	+	+	+	69.16	0.39
3		+		69.29	0.52
4	+			69.78	1.01
5				70.23	1.46
6	+		+	70.33	1.56
7		+	+	70.86	2.09
8			+	71.70	2.93

WAIC = widely applicable information criterion; ΔWAIC = difference in WAIC value relative to the minimum WAIC value; + = variable included in the hierarchical species sensitivity distribution models.

**Table 2:** The posterior medians (95% Bayesian credible intervals) of individual parameters in the hierarchical species sensitivity distribution model with the smallest widely applicable information criterion value

Parameter	Median (2.5th percentile, 97.5th percentile)
*α*	3.20 (2.23, 4.19)
*β*_size_	–0.21 (–0.62, 0.21)
*β*_media_	–0.95 (–2.20, 0.30)
*σ*	0.72 (0.48, 1.22)
*σ*_Ref_	0.92 (0.42, 1.62)

*β*_size_ and *β*_media_ = coefficients for the predictor variables particle size and type of medium (marine vs. freshwater), respectively. See the text for details about the parameters.

### Estimated HC5 values

**Figure 1:**
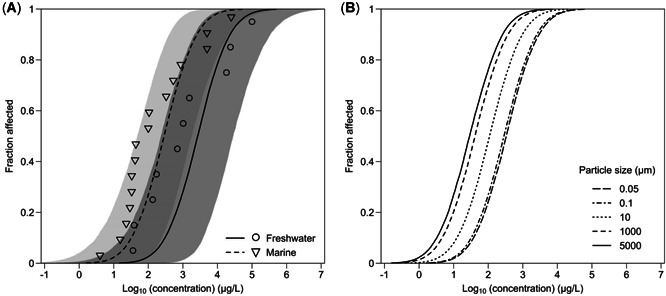
(**A**) Species sensitivity distribution (SSD) curves for nano‐ and microplastic (NMP) particles estimated using the Hamiltonian Monte Carlo samples of the best hierarchical SSD model in freshwater (solid black line, posterior median; dark gray area, 95% credible interval) and marine (black dashed line, posterior median; light gray area, 95% credible interval) environments. Only for illustration purposes, SSD plots of the chronic lowest‐observed‐effect concentrations are shown separately for freshwater (open circles) and marine (open downward triangles) media. The plastic particle size was fixed at 0.1 μm for this illustration (the lower size limit of the definition for microplastics). (**B**) The SSD curves for different NMP particle sizes (0.05, 0.1, 10, 1000, and 5000 μm) in a marine environment using the posterior medians of the parameters of the best model. Note that because the maximum particle size was 315 μm in the data set analyzed, the SSD curves for the particle sizes of 1000 and 5000 μm were extrapolated using the best model.

Based on the HMC samples of individual parameters of the minimum WAIC HSSD model, we obtained the SSD curves (Figure 1) and the posterior distributions of HC5 values for NMPs in freshwater and marine environments (Table 3). The estimated HC5 values differed by a factor of 10 depending on the type of medium; the HC5 values were 166.0 μg/L (95% credible interval, 7.6–2344.9 μg/L) and 17.6 μg/L (1.3–147.3 μg/L) in freshwater and marine environments, respectively, when the plastic particle size was assumed to be 0.1 μm (i.e., the lower size limit based on the definition of microplastics). Similarly, the SSD curves derived from posterior medians differed by a factor of approximately 10 between those based on NMPs with particle sizes of 0.05 and 5000 μm (Figure 1B), that is, within the ranges of the NMP categories. It should be noted that the HSSD modeling results for particle sizes of 1000 and 5000 μm were extrapolations because of the size range of the data sets used. The posterior median of HC5 values for NMPs with particle sizes of 0.05–5000 μm were 18.3–187.9 and 1.8–20.2 μg/L in freshwater and marine environments, respectively, although their 95% credible intervals largely overlapped.

**Table 3:** The posterior medians (95% Bayesian credible intervals) of hazardous nano‐ and microplastic concentrations for 5% of species derived from the species sensitivity distribution curves using the Hamiltonian Monte Carlo samples of the best model^a^

Medium type	Particle size (μm)	HC5 (μg/L)
Freshwater	0.05	187.9 (8.0–2978.3)
	0.1	166.0 (7.6–2344.9)
	10	62.6 (2.6–899.5)
	1000	25.3 (0.3–896.0)
	5000	18.3 (0.1–1031.0)
Marine	0.05	20.2 (1.4–192.9)
	0.1	17.6 (1.30–147.3)
	10	6.7 (0.5–64.5)
	1000	2.5 (0.1–81.1)
	5000	1.8 (<0.1–104.5)

^a^For the HC5 estimation, we did not consider the reference‐level random effects (see the text for more details.

HC5 = hazardous concentration for 5% of species.

Despite some caveats, our results suggest that failing to take these factors into account when deriving SSDs may result in HC5 estimates that depend on effect data used (e.g., the proportion of freshwater or marine effect concentrations) and thereby can be difficult to rigorously compare and interpret. For example, by combining effect data obtained in freshwater and marine media, Besseling et al. (2019) estimated HC5 values (95% confidence intervals) to be 5.4 μg/L (0.93–31 μg/L) and 1.7 μg/L (0.086–33 μg/L) for nano‐ and microplastics, respectively. Those estimates were approximately equivalent to our estimates of HC5 for particle sizes of 10–5000 μm in marine environments even though our estimates were based on their data sets; however, we removed and processed several effect data for practical reasons (see *Materials and Methods* for details). Compared to Besseling et al. (2019), the credible intervals for the HC5 values estimated in the present study were often wider (Table 3). This is likely because our HSSD had more parameters to be estimated but generally used the same data set. Consequently, applying our HSSD modeling to larger data sets would be valuable to narrow the ranges of credible intervals.

### Influence of test type of medium

Our results of HSSD modeling suggest that chronic LOECs obtained from tests with marine media were on average lower than those with freshwater media, although we could not strictly discriminate whether this result was caused by the difference in the media tested and/or in the species tested. For chemicals, on average, such systematic differences in effect concentrations between freshwater and saltwater species were not observed (see de Zwart, 2002; Wheeler et al., 2002, 2014). According to a previous study that derived SSDs for nanoplastics (Yang & Nowack, 2020), the HC5 value estimated based on the full data set obtained in marine media (1.3 μg/L) was lower than that obtained in freshwater media (71 μg/L), but values were comparable after removing data measured in the presence of NaN_3_. It is not possible to identify the underlying reasons because of the limited availability of data. Thus, more effect data should be acquired to clarify the difference in effect concentrations of NMPs in freshwater and marine media; particularly, comparing effect concentrations obtained for the same species that can be tested in both freshwater and marine media (e.g., Japanese medaka [*Oryzias latipes*]) would allow the influence of test media to be further investigated. In addition, in the analyzed data set, the salinity of the seawater used varied among the studies and was not reported in some of the studies. Although the importance of water quality parameters such as salinity for predicting effect concentrations of NMPs is still uncertain, we recommend reporting the details of the water quality in the test media.

### Influence of plastic particle size

Evidence from the literature indicates that smaller plastic particles have lower effect concentrations than larger particles, which is likely related to the ease of accumulation and longer retention time of smaller particles in digestive organs compared to larger particles, as well as their larger relative surface area (Jacob et al., 2020; Jeong et al., 2017; Li et al., 2020). In addition, smaller‐sized nanoplastics can penetrate the cell membranes and exhibit ecotoxicity to aquatic organisms (Shen et al., 2019). However, the results of our HSSD modeling did not support the positive relationship. In our HSSD modeling, we used the log_10_‐transformed particle diameter to examine the influence of particle size on the effect concentrations, and we did not consider the relative size of NMP particles for each test species. However, when we used the ratio of particle size to body size of each test species, we still found a negative association between this particle size variable and effect concentrations (Supporting Information, Tables S2 and S3). Some empirical studies have not observed particle size–dependent toxicity (e.g., Choi et al., 2020; Niu et al., 2021). Furthermore, Yang and Nowack (2020) concluded that nanoplastics are likely less hazardous than microplastics based on the comparison of several published PNECs. Again, there are still issues to overcome before we can accurately account for size‐dependent bioavailability and toxicity in estimating HSSDs (see Koelmans et al. [2020] for the correction method to estimating the bioavailable fraction), but our results regarding the influence of particle size in the HSSD modeling support the conclusion by Yang and Nowack (2020).

### Influence of polymer type

The predictor variable polymer type was not included in the best model, suggesting that the difference in chronic LOECs between PS and other polymer types was not substantial in the data set analyzed. Although a few previous studies support this result (e.g., Adam et al., 2019), caution is required when interpreting this result. First, it may have been difficult to detect substantial differences in effect concentrations between PS and other polymer types because most of the polymer types available in the data set (22 of 26) were PS. Second, note that polymer type was included as a predictor variable in the second‐best model (Table 1). Based on the parameter *β*_polymer_ in the second‐best model, the chronic LOECs for PS were estimated to be lower (i.e., more toxic) than those for other polymer types (Supporting Tables S1 and S4). Given the importance of differentiating between physical effect and chemical toxicity (Zimmermann et al., 2020), more detailed research on differences in effect concentrations among polymer types is warranted.

### Influence of reference‐specific unmodeled factors

We incorporated the reference‐level random effects (parameter *r*) in the HSSD modeling to describe the variations in effect concentrations due to reference‐specific unmodeled factors. These unmodeled factors include differences in physicochemical conditions (e.g., the presence or absence of NMP particle preprocessing including removal of sodium azide stabilizer (Besseling et al., 2019; Yang & Nowack, 2020) and biological conditions (e.g., developmental stage and origin of organisms) in tests. Without incorporating random effects in the estimation of HC5, the posterior medians of HC5 were 166.0 and 17.6 μg/L for plastic particles with a size of 0.1 μm in freshwater and marine environments, respectively. On the other hand, when the influence of *σ*_Ref_ was accounted for in the estimation of HC5 (i.e., by adding values randomly generated from the Gaussian distribution with mean 0 and standard deviation *σ*_Ref_ in the calculation of *μ*), the posterior medians of the HC5 largely did not change (141.6 and 15.2 μg/L, respectively), but the 95% credible intervals of HC5 increased approximately by a total of 2 orders of magnitude (1.1–39,370.1 and 0.1–3980.1 μg/L in freshwater and marine media, respectively; see Table 3 and Supporting Information, Table S5, for more details). Although such uncertainties have been noted (Besseling et al., 2019) or partly addressed (Yang & Nowack, 2020) in previous studies, our study has provided the first quantitative assessment of the magnitude of the influence of the unmodeled factors on HC5 estimates. As noted, the magnitude of credible intervals for HC5 values in the HSSDs should have been affected largely by the relatively small sample size of the analyzed data set, so our quantitative conclusion regarding “a total of 2 orders of magnitude” should be interpreted with caution. Nevertheless, our study still emphasizes the importance of addressing such factors in the SSD derivation to reduce estimation uncertainties.

### Implications for future model development and application

To our knowledge, this is the first study that has illustrated HSSD modeling for NMP particles and estimated the HC5 values by quantitatively and simultaneously considering the influences of NMP properties (particle size and polymer type) and type of medium, despite the use of limited effect data. In contrast to the SSD estimation for single chemicals in general, NMP particles are mixtures of particles with diverse properties. The HSSD approach illustrated in the present study would be particularly useful for such situations because it can directly incorporate the influences of diverse properties into the SSD derivation. Similar approaches can be applied to certain chemical groups such as pesticides. In the present study, geometric means were used for the HSSD estimation if multiple effect concentrations were available under the same test conditions. However, as was modeled in the probabilistic SSD approach (Wigger et al., 2020), the HSSD approach can directly use these effect concentrations in modeling by considering the intraspecies variations in effect concentrations, though we could not adopt such modeling here, largely because of the limited availability of effect data used in the present study. Given that knowledge about the ecotoxicity of NMP particles has been rapidly growing, further work on the application of HSSD models to larger (preferably quality‐assured) data sets should help guide and improve the hazard assessment in ecological risk assessments for NMPs.

## Supporting Information

The Supporting Information is available on the Wiley Online Library at https://doi.org/10.1002/etc.5295.

## Disclaimer

The authors declare that they have no conflicts of interest.

## Author Contributions Statement

Conceptualization, data curation, formal analysis, methodology, software, visualization, writing—original draft, writing—review and editing: Kazutaka M. Takeshita. Conceptualization, data curation, formal analysis, funding acquisition, methodology, project administration, writing—original draft, writing—review and editing: Yuichi Iwasaki. Methodology, writing—review and editing: Thomas M. Sinclair. Methodology, writing—review and editing: Takehiko I. Hayashi. Funding acquisition, project administration, writing—review and editing: Wataru Naito.

## Supporting information

This article contains online‐only Supporting Information.

Supporting information.

Supporting information.

## Data Availability

All data sets supporting this article have been included in the Supporting Data. Data, associated metadata, and calculation tools are also available from the corresponding author (yuichiwsk@gmail.com).
