# Supplementary material for: Illustrating a Species Sensitivity Distribution for Nano‐ and Microplastic Particles Using Bayesian Hierarchical Modeling
Source: Environ Toxicol Chem. 2022 Feb 28;41(4):954–60. doi: 10.1002/etc.5295 (PMC9314701; doi:10.1002/etc.5295)
Supplement: Supplementary file 1 — Supporting information. [file ETC-41-954-s001.docx]

Supplementary Material for “Illustrating a species sensitivity distribution for nano- and microplastic particles by using Bayesian hierarchical modeling”

**List of materials**

R and Stan program code

Figure S1

Table S1

Table S2

Table S3

Table S4

Table S5

An R and Stan program code for a hierarchical species sensitivity distribution model. The model with all the three predictor variables is provided as an example.

#Read effect concentration dataset

library(openxlsx)

d <- read.xlsx("Supplemental_Data.xlsx", sheet = "Dataset")

list.data <- list(

N = nrow(d),

R = max(as.numeric(as.factor(d$Reference))),

Log10_tox = log10(d$LOEC),

Log10_size = log10(d$Ave_size),

Media_type = as.numeric(d$Media_type_r == "Marine"),

Polymer_type = as.numeric(d$Polymer_type_r == "PS"),

Reference = as.numeric(as.factor(d$Reference))

)

#Load package

library(rstan)

rstan_options(auto_write = T); options(mc.cores = parallel::detectCores())

cat("data {

int<lower = 0> N; // number of data

int<lower = 0> R; // number of references

real Log10_tox[N]; // log_10_-transformed chronic LOEC

real Log10_size[N]; // log_10_-transformed plastic-particle size

int<lower = 0, upper = 1> Media_type[N]; // binary-dummy variable representing type of medium (freshwater: 0, marine: 1)

int<lower = 0, upper = 1> Polymer_type[N]; // binary-dummy variable representing polymer type (polymer types other than PS: 0, PS: 1)

int<lower = 0> Reference[N]; // variable to identify the reference

}

parameters {

real alpha;

real beta[3];

real eff_ref[R];

real<lower = 0> sigma;

real<lower = 0> sigma_ref;

}

transformed parameters {

real mu[N];

for (n in 1:N) {

mu[n] = alpha + beta[1] * Log10_size[n] + beta[2] * Media_type[n] + beta[3] * Polymer_type[n] + eff_ref[Reference[n]];

}

}

model {

for (n in 1:N) {

Log10_tox[n] ~ normal(mu[n], sigma);

}

for (r in 1:R)

eff_ref[r] ~ normal(0, sigma_ref);

}", "\n", file = "hssd.stan")

stanmodel <- stan_model(file = 'hssd.stan')

#Start sampling

fit <- sampling(

stanmodel,

data = list.data,

pars = c('alpha', 'beta', 'sigma', 'eff_ref', 'sigma_ref'),

chains = 3,

iter = 30000,

warmup = 20000,

thin = 10,

control = list(adapt_delta = 0.9),

seed = 1010

)

#save(fit, file = "res.Rdata")

**Supplemental figure**


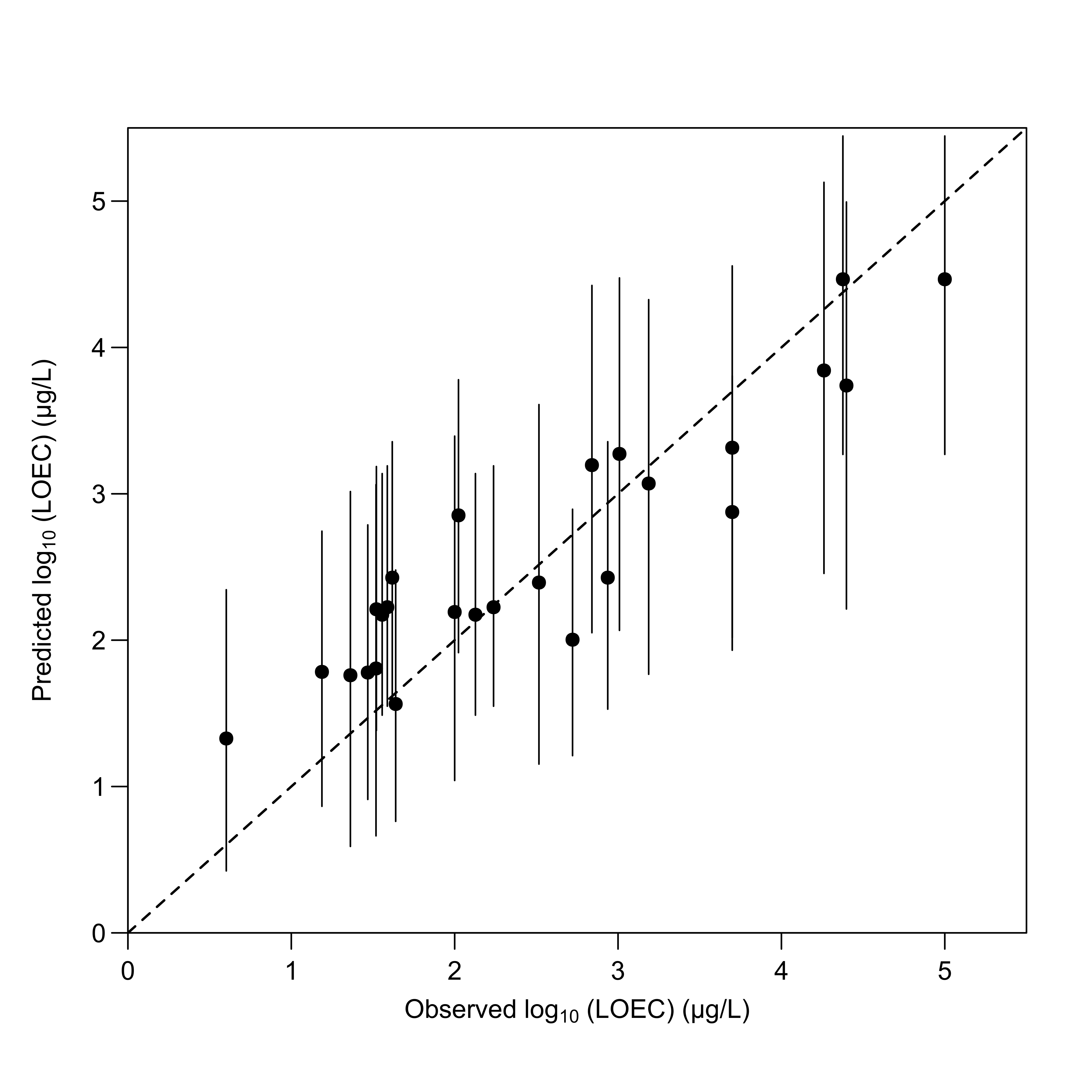


**Figure S1:** Relationship between the observed and predicted chronic LOECs. Predicted values were estimated from the hierarchical species sensitivity distribution model with the smallest widely applicable information criterion value (see the text for more details). For predicted values, the posterior medians (black dots) and 95% Bayesian prediction intervals (bars) are shown. The dashed black line indicates the equality line.

**Supplemental tables**

**Table S1.** The posterior medians (95% Bayesian credible intervals) of individual parameters in the hierarchical species sensitivity distribution model with the second minimum widely applicable information criterion value

| Parameter | Median (2.5th percentile, 97.5th percentile) |
| --- | --- |
| *α* | 3.92 (2.41, 5.36) |
| *β*_size_ | –0.40 (–0.90, 0.10) |
| *β*_media_ | –0.81 (–2.09, 0.34) |
| *β*_polymer_ | –1.09 (–2.82, 0.64) |
| *σ* | 0.73 (0.49, 1.21) |
| *σ*_Ref_ | 0.87 (0.31, 1.60) |

*β*_size_, *β*_media_, and *β*_polymer_ are the coefficients for the predictor variables particle size, type of medium (marine vs. freshwater), and polymer type (PS vs. polymer types other than PS), respectively. See the text for more details.

**Table S2.** The posterior medians (95% Bayesian credible intervals) of individual parameters in the hierarchical species sensitivity distribution model with all predictor variables. This model differs from that in the main document in that it uses the ratio of particle size to body size of each test species instead of the value of the particle size itself (see Table S3 for the body size value of each species).

| Parameter | Median (2.5th percentile, 97.5th percentile) |
| --- | --- |
| *α* | 3.25 (1.65, 4.83) |
| *β*_size_ | –0.008 (–0.43, 0.24) |
| *β*_media_ | –0.75 (–2.13, 0.56) |
| *β*_polymer_ | –0.42 (–2.06, 1.38) |
| *σ* | 0.79 (0.52, 1.32) |
| *σ*_Ref_ | 0.94 (0.19, 1.76) |

*β*_size_, *β*_media_, and *β*_polymer_ are the coefficients for the predictor variables particle size, type of medium (marine vs. freshwater), and polymer type (PS vs. polymer types other than PS), respectively. See the text for more details.

**Table S3.** Body size values and references of the species in the dataset used for hierarchical species sensitivity distribution modeling shown in Table S2. *Crassostrea gigas*, for which we could not find information about the representative body size value, was not included in the analysis shown in Table S2.

| Species | Body size | Reference |
| --- | --- | --- |
| *Artemia franciscana* | 0.9 mm | Kokalj et al., 2018 |
| *Brachionus koreanus* | 0.172 mm | Kang et al., 2019 |
| *Calanus helgolandicus* | 2.24 mm | Cole et al., 2015 |
| *Crassostrea gigas* | - | - |
| *Daphnia magna* | 5 mm | Koelmans et al., 2020 |
| *Dunaliella tertiolecta* | 10 μm | Throndsen, 1996 |
| *Hyalella azteca* | 7.8 mm | Schmitz and Scherrey, 1983 |
| *Lemna minor* | 3 mm | Pistone et al., 1999 |
| *Paracentrotus lividus* | 6 cm | Sala, 1997 |
| *Pseudokirchneriella subcapitata*  (*Raphidocelis subcapitata*) | 11 μm | OECD, 2011 |
| *Scenedesmus obliquus* | 7.5 μm | National Institute for Environmental Studies, 2020 |
| *Skeletonema costatum* | 10 μm | Gu et al., 2012 |
| *Thamnocephalus platyurus* | 1120 μm | Kurmayer and Jüttner, 1999 |
| *Tigriopus japonicus* | 984 μm | Raisuddin et al., 2007 |
| *Tripneustes gratilla* | 93 μm | Kaposi et al., 2014 |
| *Xenopus laevis* | 78.5 mm | Katbamna et al., 2006 |

**References for Table S3**

Cole, M., Lindeque, P., Fileman, E., Halsband, C., & Galloway, T. S. (2015). The impact of polystyrene microplastics on feeding, function and fecundity in the marine copepod *Calanus helgolandicus*. *Environmental Science & Technology*, 49, 1130–1137. https://doi.org/10.1021/es504525u

Gu, H., Zhang, X., Sun, J., & Luo, Z. (2012). Diversity and seasonal occurrence of *Skeletonema* (Bacillariophyta) species in Xiamen Harbour and surrounding seas, China. *Cryptogamie Algologie*, 33, 245–263. https://doi.org/10.7872/crya.v33.iss3.2012.245

Kang, H. M., Lee, J. S., Lee, Y. H., Kim, M. S., Park, H. G., Jeong, C. B., & Lee, J. S. (2019). Body size-dependent interspecific tolerance to cadmium and their molecular responses in the marine rotifer *Brachionus* spp. *Aquatic Toxicology*, 206, 195–202. https://doi.org/10.1016/j.aquatox.2018.10.020

Kaposi, K. L., Mos, B., Kelaher, B. P., & Dworjanyn, S. A. (2014). Ingestion of microplastic has limited impact on a marine larva. *Environmental Science & Technology*, 48, 1638–1645. https://doi.org/10.1021/es404295e

Katbamna, B., Brown, J. A., Collard, M., & Ide, C. F. (2006). Auditory brainstem responses to airborne sounds in the aquatic frog *Xenopus laevis*: correlation with middle ear characteristics. *Journal of Comparative Physiology A*, 192, 381–387. https://doi.org/10.1007/s00359-005-0076-3

Koelmans, A. A., Redondo-Hasselerharm, P. E., Mohamed Nor, N. H., & Kooi, M. (2020). Solving the nonalignment of methods and approaches used in microplastic research to consistently characterize risk. *Environmental Science & Technology*, 54, 12307–12315. https://doi.org/10.1021/acs.est.0c02982

Kokalj, A. J., Kunej, U., & Skalar, T. (2018). Screening study of four environmentally relevant microplastic pollutants: uptake and effects on *Daphnia magna* and *Artemia franciscana*. *Chemosphere*, 208, 522–529. https://doi.org/10.1016/j.chemosphere.2018.05.172

Kurmayer, R., & Jüttner, F. (1999). Strategies for the co-existence of zooplankton with the toxic cyanobacterium *Planktothrix rubescens* in Lake Zurich. *Journal of Plankton Research*, 21, 659–683. https://doi.org/10.1093/plankt/21.4.659

National Institute for Environmental Studies. (2020). NIES collection Microbial Culture Collection. Tsukuba, Ibaraki, Japan. National Institute for Environmental Studies. Retrieved April 19, 2021, from https://mcc.nies.go.jp/strainList.do?lang=en&strainId=2672

OECD. (2011). Test No. 201: Freshwater alga and cyanobacteria, growth inhibition test. *OECD Guidelines for the Testing of Chemicals, section 2*. https://doi.org/10.1787/9789264069923-en

Pistone, G., Allinson, G., Stagnitti, F., & Colville, S. (1999). Effect of selenium on the growth of *Lemna minor*. *Toxicological & Environmental Chemistry*, 71, 271–277. https://doi.org/10.1080/02772249909358799

Raisuddin, S., Kwok, K. W. H., Leung, K. M. Y., Schlenk, D., & Lee, J. S. (2007). The copepod *Tigriopus*: a promising marine model organism for ecotoxicology and environmental genomics. *Aquatic Toxicology*, 83, 161–173. https://doi.org/10.1016/j.aquatox.2007.04.005

Sala, E. (1997). Fish predators and scavengers of the sea urchin *Paracentrotus lividus* in protected areas of the north-west Mediterranean Sea. *Marine Biology*, 129, 531–539. https://doi.org/10.1007/s002270050194

Schmitz, E. H., & Scherrey, P. M. (1983). Digestive anatomy of *Halella azteca* (Crustacea, Amphipoda). *Journal of Morphology*, 175, 91–100. https://doi.org/10.1002/jmor.1051750109

Throndsen, J. (1996). The planktonic marine flagellates. In C. R. Tomas (Eds.), *Identifying marine phytoplankton* (pp. 591–730). Academic Press.

**Table S4.** The posterior medians (95% Bayesian credible intervals) of hazardous plastic concentrations for 5% of species (HC5) derived from the species sensitivity distribution curves using the Hamiltonian Monte Carlo samples of the model with the second minimum widely applicable information criterion value. For the HC5 estimation, we did not consider the reference-level random effects (see the text for more details). The plastic particle size was fixed at 0.1 μm for this estimation (the lower limit of the definition for microplastics).

| Media type | Polymer type | HC5 (μg/L) |
| --- | --- | --- |
| Freshwater | PS | 94.0 (4.8–1521.4) |
|  | Others | 1172.6 (13.9–56574.2) |
| Marine | PS | 14.4 (1.0–119.8) |
|  | Others | 180.7 (1.5–10328.6) |

**Table S5.** The posterior medians (95% Bayesian credible intervals) of hazardous nano- and microplastic concentrations for 5% of species (HC5) derived from the species sensitivity distribution curves using the Hamiltonian Monte Carlo samples of the best model. For the HC5 estimation, we considered the reference-level random effects (see the text for more details).

| Media type | Particle size (μm) | HC5 (μg/L) |
| --- | --- | --- |
| Freshwater | 0.05 | 160.4 (1.2–52652.5) |
|  | 0.1 | 141.6 (1.1–39370.1) |
|  | 10 | 55.9 (0.3–12869.8) |
|  | 1000 | 21.4 (0.1–8301.7) |
|  | 5000 | 15.6 (<0.1–7276.4) |
| Marine | 0.05 | 17.4 (0.1–5030.5) |
|  | 0.1 | 15.2 (0.1–3980.1) |
|  | 10 | 5.9 (<0.1–1481.0) |
|  | 1000 | 2.3 (<0.1–929.6) |
|  | 5000 | 1.7 (<0.1–939.7) |
